# Postoperative health-related quality of life in children with congenital heart disease: a short-term follow-up study

**DOI:** 10.1186/s13019-023-02110-x

**Published:** 2023-01-11

**Authors:** Wang-Sheng Dai, Wen-Hao Lin, Shi-Hao Lin, Qiang Chen, Hua Cao

**Affiliations:** Department of Cardiac Surgery, Fujian Children’s Hospital (Fujian Branch of Shanghai Children’s Medical Center), College of Clinical Medicine for Obstetrics & Gynecology and Pediatrics, Fujian Medical University, Fuzhou, China

**Keywords:** Congenital heart disease, Health-related quality of life, Surgical treatment

## Abstract

**Objective:**

This study aimed to explore changes in health-related quality of life in children with congenital heart disease from pre-surgery to 6 months after surgery.

**Methods:**

A total of 87 children aged 2–12 years who underwent cardiac surgery in a provincial hospital in China from January 2021 to June 2021 were selected. After 6 months, the quality of life of all children was retrospectively analyzed. The Chinese version of the Pediatric Quality of Life Inventory 4.0 Scale was used to assess the quality of life of children before and after surgery.

**Results:**

Parents of 85 children and 33 children aged 5–12 years completed the questionnaires. After surgical treatment, the quality of life scores reported by parents of children of all ages were significantly higher than those before surgery, the *P *value < 0.05; the self-evaluated quality of life scores of children of different ages were significantly higher than those before surgery, the *P *value < 0.05.

**Conclusion:**

Surgical treatment can improve the health-related quality of life of children with congenital heart disease.

## Introduction

The incidence of congenital heart disease (CHD) has remained high in recent years, and it has become the most common birth defect [1], becoming the leading cause of death and disability in infants and young children. With the improvement of the diagnosis and treatment technology of CHD and the success rate of cardiac surgery, surgical and interventional treatment can enable at least 85% of children with CHD to survive into adulthood [2]. In the traditional sense, mortality and cardiac function are used to evaluate the effectiveness of treatment and rehabilitation. However, with the increasing understanding of health, people realize that the current indicators cannot fully evaluate the effectiveness of rehabilitation [3]. Quality of life assessment is a comprehensive assessment of a person's health in three aspects: physical, mental and social activities, and becomes an important indicator to assess the physical and mental health of children. It reflects the children's cognition and satisfaction with their current life conditions. A systematic review and meta-analysis showed that health-related quality of life was worse in postoperative patients with CHD versus healthy controls in all domains [4]. Therefore, it is important to pay attention to the postoperative quality of life of children with CHD to understand their health level and to improve their quality of life.

Children with CHD have poorer blood transport and weaker gastrointestinal function due to heart insufficiency, resulting in impaired nutritional intake. At the same time, the energy consumption of these children is greater than that of normal children of the same age, leading to insufficient nutritional supply and lagging growth [5]. Postoperative activities of children are restricted, and postoperative wounds will also affect aesthetics, which can affect the future life and learning of the children. These also affect their quality of life to a certain extent. Studies have found that the overall postoperative quality of life of children with CHD is lower than that of children without CHD of the same age [6]. Therefore, it is worth paying special attention to the quality of life of this group of children. The purpose of this study is to investigate changes in quality of life in children with CHD from pre-surgery to 6 months after surgery.

## Information and methods

### General information

The clinical data of 87 children with CHD who were admitted to our hospital from January 2021 to June 2021 were collected, and the health-related quality of life before and after the operation was retrospectively analyzed. Eighty-five participants completed the study, including 52 aged 2–4 years, 21 aged 5–7 years, and 12 aged 8–12 years. The inclusion criteria were as follows: 1. CHD was diagnosed and treated surgically; 2. postoperative echocardiography showed satisfactory correction of cardiac malformations; 3. there were no serious complications in other organs during the perioperative period; 4. the children and their parents had normal understanding and expression skills; 5. the children and their parents agreed to participate in this study and signed an informed consent form. The exclusion criteria were as follows: 1. serious complications and hemodynamic instability occurred during the perioperative period; 2. the children and their parents refused to participate in this study.

### Methods

This was a retrospective study. All research procedures had been approved by the ethics committee of Fujian Medical University and were conducted in accordance with the Helsinki declaration. Participants and their parents would be informed of the research process in detail and informed consent was obtained from the parents. Researchers collected relevant information of the children, such as gender, age, type of disease, and so on. On the day before surgery, researchers asked questions to family members and children aged 5–12 years to complete children’ health-related quality of life scores. At the children’ follow-up visit 6 months after surgery, researchers again asked questions to the families and children aged 5–12 years to complete the health-related quality of life scores. The tool used to assess health-related quality of life was the Pediatric Quality of Life Inventory 4.0 (PedsQL 4.0) scale. The PedsQL was completed in the waiting room or in the clinical examination room. The research team included 1 cardiac surgeon, 1 statistician, and 2 medical assistants.

### Observation index

The Pediatric Quality of Life Inventory 4.0 Scale (PedsQL4.0): it was a commonly used scale for measuring children’s quality of life, developed by the San Diego Children’s Hospital in California, USA. It was designed for children aged 2–18 years, and it was developed from a large number of healthy children and children with acute or chronic health conditions [7]. The PedsQL4.0 questionnaire had four multidimensional scales: physical function (8 items), emotional function (5 items), social function (5 items) and school function (5 items). The three total scores were the total scale score (23 items), the total physical health score (8 items) and the total psychosocial health score (15 items). The total score of each dimension was calculated as the sum of the scores of all questions in the dimension divided by the number of questions in that dimension, and the total score for the scale was calculated as the total score for all questions divided by the total number of questions. The scores for each dimension and the total scale score ranged from 0 to 100. The better the quality of life, the higher the score. PedsQL™ only had a parent report questionnaire for children aged 2–4 years, and the scales for other age groups included two scales: pediatric self-evaluation and parent report. Self-evaluation scales for children aged 8–18 and parent report scales both had a 5-point Likert scale from 0 to 4 (0, never a problem; 1, almost never a problem; 2, sometimes a problem; 3, usually a problem; 4, almost always a problem); the self-evaluation scale for younger children aged 5–7 used a simple 3-point scale from 0 to 3 (0 = not a problem at all; 1 = sometimes a problem; 3 = very difficult). For children aged 5–7 years, if they could respond verbally or by nodding or gesturing, they completed the scale with the help of their parents. After repeated evaluation and application, it had been proved that PedsQL4.0 had good reliability and validity [8]. It had been widely translated into multiple languages and had been widely used in measuring the quality of life of children with CHD.

### Sample size

The sample size was determined with PASS 15. 0. The alpha value was set at 0.05 and a power of 0.90. Based on the calculation, the resulting minimum sample size was 74 patients. Considering a 15% drop rate, we included 87 samples for the research.

### Data collection

The researchers screened 87 eligible children for the study, and 2 of them refused to participate in the study. Parents of 85 children and 33 children aged 5–12 years completed the questionnaires before and 6 months after the operation.

### Statistical analysis

SPSS 22.0 was used for statistical analysis in the study. Quantitative data were expressed as mean ± standard deviation, and a normal distribution test was carried out. The health-related quality of life scores before and after surgery did not follow a normal distribution according to a normality test. The Wilcoxon test was used to compare the differences in quality of life scores before and after surgery. *P* < 0.05 indicated that the difference was significant.

## Results

Table 1 showed the general information of all participants, such as age, gender, type of disease and so on. There were 39 cases of ventricular septal defect (VSD), 25 cases of atrial septal defect (ASD), 13 cases of patent ductus arteriosus (PDA), and 8 cases of pulmonary valve stenosis (PS) in this study.

**Table 1:** Baseline socio-demographic characteristics of participants

Characteristics	N = 85 (n%)
*Age range (years)*	
2–4	52 (61.2%)
5–7	21 (24.7%)
8–12	12 (14.1%)
*Gender*	
Male	47 (55.3%)
Female	38 (44.7%)
*Parents' education*	
Junior high school or lower	43 (50.6%)
High school	23 (27.1%)
bachelor’s degree or higher	19 (22.3%)
*Family income*	
Poor	26 (30.6%)
Median level	38 (44.7%)
Rich	21 (24.7%)
*Types of diseases*	
Ventricular septal defect	39 (45.9%)
Atrial septal defect	25 (29.4%)
Patent ductus arteriosus	13 (15.3%)
Pulmonary valve stenosis	8 (9.4%)
*NYHA*	
I/II	85 (100%)
III/IV	0 (0%)

*NYHA* New York Heart Association

As shown in Table 2, it listed the health-related quality of life scores reported by parents of children in different age groups. At 6 months after surgery, the scores of the four dimensions and the total score were significantly higher than those before the surgery, the *P* < 0.05.

**Table 2:** Comparison of health-related quality of life scores reported by parents of children in different age groups before and after surgery

Item	2–4 year (n = 52)		5–7 year (n = 21)		8–12 year (n = 12)	
	Before	After	Before	After	Before	After
Physical functioning	69.23 ± 11.43	81.05 ± 8.83*	70.56 ± 12.45	82.45 ± 9.38*	71.48 ± 13.08	83.29 ± 13.05*
Emotional functioning	70.29 ± 10.77	80.39 ± 9.96*	70.23 ± 11.97	81.95 ± 8.59*	71.23 ± 11.38	81.22 ± 10.38*
Social functioning	70.58 ± 10.46	82.14 ± 10.24*	72.46 ± 11.35	83.38 ± 11.52*	70.48 ± 11.02	84.81 ± 9.92*
School functioning	70.06 ± 13.93	80.68 ± 10.56*	71.84 ± 11.38	82.96 ± 10.92*	74.38 ± 12.94	85.35 ± 9.45*
Total score	70.31 ± 9.84	81.46 ± 8.07*	71.65 ± 8.26	82.77 ± 7.55*	72.95 ± 8.80	84.03 ± 8.64*

Before: before surgery, After: 6 months after surgery

*p < 0.05 compared with before surgery

As shown in Table 3, it listed the self-evaluated health-related quality of life scores of children in different age groups. At 6 months after surgery, the scores of the four dimensions and the total score were significantly higher than those before the surgery, and the P < 0.05. There was no significant difference between the self-evaluated quality of life scores of children aged 5–7 years and those of children aged 8–12 years, but the scores of children aged 5–7 years were slightly higher than those of children aged 8**–**12 years.

**Table 3:** Comparison of self-rated health-related quality of life scores for children in different age groups before and after surgery

Item	5–7 year (n = 21)		8–12 year (n = 12)	
	Before surgery	6 months after surgery	Before surgery	6 months after surgery
Physical functioning	71.48 ± 13.08	83.29 ± 13.05*	70.56 ± 12.45	82.45 ± 9.38*
Emotional functioning	71.23 ± 11.38	81.22 ± 10.38*	70.23 ± 11.97	81.95 ± 8.59*
Social functioning	70.48 ± 11.02	84.81 ± 9.92*	72.46 ± 11.35	83.38 ± 11.52*
School functioning	74.38 ± 12.94	85.35 ± 9.45*	71.84 ± 11.38	82.96 ± 10.92*
Total score	72.76 ± 9.37	84.59 ± 8.04*	71.38 ± 8.75	83.01 ± 7.96*

*p < 0.05 compared with before surgery

## Discussion

Quality of life is a multidimensional concept. The World Health Organization defines it as being based on the individual's value system, cultural background and one's own subjective feelings about life, and affected by the individual's expectations, standards, goals, and attention [9]. In the "Research on the Quality of Life of Special Populations", the research on the quality of life of children is ranked fifth [10]. The quality of life of children with CHD is determined in a variety of ways, including the children's psychological, life and social function status. Unlike the quality of life assessment methods for adults, because the growth and cognitive abilities of younger children are not yet perfect [11], medical staff can only make scientific and objective assessments of their physiological conditions, but parents can make extensive observations of their children's activities, which are more effective in evaluating their emotional and thinking conditions [12].

Children with CHD have poor blood transport efficiency due to cardiac insufficiency [13], resulting in weak gastrointestinal function and inadequate nutritional intake. In addition, such children have high energy consumption [14] and the nutritional demand is greater than the supply, leading to lagging growth and development and affecting their appearance. The study of Argent showed that the growth and development of children with CHD significantly lagged behind that of normal children [15]. The better the children’s development level, the better the quality of life. Therefore, it is necessary to increase postoperative nutritional support for children to improve their growth and development and to improve their quality of life.

Surgery is considered to be the most effective modality in the treatment of CHD [16]. After surgical treatment, Children with CHD have improved cardiac function and quality of life [17]. The results of this study showed that the parent-reported health-related quality of life scores and child self-rated quality of life scores of children in different age groups significantly improved after surgery, suggesting the success of surgical treatment. It also showed that the prognosis of children after surgical treatment was ideal, and the quality of life was improved.

In this study, there were no significant differences in the self-rated quality of life scores of children in different age groups, but the scores were slightly higher in the 5–7 years old group than in the 8–12 years old group. The reason is that there is a certain correlation between the quality of life and the level of children's growth and development [18]. 8–12 year olds have increased analytical and comprehension skills and have made significant psychological transitions during this period. Children's self-awareness increases with age [19], and they have higher quality of life requirements, so the self-rated quality of life scores are lower than in the lower age groups. In addition, the surgical level of surgeons is also one of the factors that affect the score [20]. Surgical techniques are advancing and aggressive surgical treatment in the early stages of the disease can lead to a higher quality of life [21]. At the same time, the quality of life is also affected by social and parental factors. The prognosis of children with a family income that can afford good treatment conditions and provide good nutritional conditions and a comfortable and safe living environment is better [22, 23]. Access to good social health maintenance services is also an important factor in improving the quality of life of children after surgery [24].

In adults, increasing the amount of exercise can improve their quality of life after CHD surgery [25]. Therefore, it is possible to explore the relationship between increased exercise and the quality of life of children with CHD after surgery [26, 27]. We should pay attention to the life, study, and work situation of this population, and promptly and effectively interventions with the children and their parents after the operation should be implemented to strengthen the children's coping ability and improve their quality of life.

With the continuous development of science, technology and medicine, more and more children with CHD will survive after surgery. At present, we have a basic understanding of the current situation of quality of life of postoperative children with CHD, but the lack of long-term follow-up observation makes the research results not deep and comprehensive. Therefore, it is necessary to carry out a long-term follow-up of the children and investigate the current situation of the children's life at different time periods after surgery in order to truly reflect the quality of life and its change process after surgery [28]. Meanwhile, we should strengthen the multicenter and multidisciplinary cooperation, and strive to improve the quality of life of children with CHD after surgery, so that they can better face various challenges in the future.

This study has some shortcomings. First of all, it was a retrospective study rather than a randomized controlled study. Therefore, the cases had certain deviations, but the results still have certain clinical significance. Secondly, this was a single-center study with a relatively small sample size and a follow-up time of only 6 months. Finally, this study was limited to specific patients who underwent surgery for CHD, other patients may have different results. Therefore, a multicenter, prospective and long-term study will be conducted in the future to determine the results of the study.

## Conclusion

In addition to correcting the congenital malformations of children with CHD, surgical treatment also has a positive significance for improving their health-related quality of life. Therefore, children with CHD should be diagnosed and treated as early as possible in an effort to improve their health-related quality of life.

## Data Availability

The data sets used and/or analyzed during the current study are available from the first author or the corresponding author on reasonable request.

## Abbreviation

CHD: Congenital heart disease
